# Ageing characteristics of bone indicated by transcriptomic and exosomal proteomic analysis of cortical bone cells

**DOI:** 10.1186/s13018-019-1163-4

**Published:** 2019-05-10

**Authors:** Chenyang Zhang, Shuyu Xu, Shufan Zhang, Mengmeng Liu, Haiming Du, Ruinan Sun, Bo Jing, Yao Sun

**Affiliations:** 1 Department of Oral Implantology, School and Hospital of Stomatology, Tongji University, Shanghai Engineering Research Center of Tooth Restoration and Regeneration, 399 Middle Yanchang Road, Shanghai, 200072 China; 2 Department of Biology, Southern University of Science and Technology, Shenzhen, China

**Keywords:** Bone ageing, Gene expression profiling, Exosome, OLCS

## Abstract

**Background:**

Degenerative changes in the skeleton play an important role in ageing. As the foremost sensors and orchestrators of bone remodelling, osteocytes contribute significantly to the health of the skeleton. Embedded in a mineralized bone matrix, the osteocyte network and the surrounding lacunar canaliculae work together as a functional syncytium—the osteocytic lacunar-canalicular system (OLCS). However, changes in the OLCS during ageing and related mechanisms cannot be fully understood by using traditional histological analysis.

**Methods:**

To link the phenotypes of aged osteocytes and their functional changes during ageing, we analysed the changes in the gene expression profiles of bone cells and the proteomic profiles of OLCS exosomes derived from aged and young cortical bone.

**Results:**

Gene Ontology (GO) and Kyoto Encyclopedia of Genes and Genomes (KEGG) analysis of differentially expressed genes (DEGs) suggested that a decline in cell energy metabolism and an increased level of the proinflammatory state are major characteristics of bone ageing. Moreover, some DEGs were key regulators of bone mechanical sensation and bone remodelling, which are indicative of reduced bone-specific function with age. Further, the identified proteins in OLCS exosomes showed potential changes in the secretory function bone. Compared with young controls, the decreased functional proteins in aged OLCS exosomes were enriched mainly in GO terms that included regulating bone development and remodelling, cell-matrix adhesion, and cell clearance and homeostasis. Notably, several functions of exosomal proteins of the aged group revealed potential new roles, such as regulating innate and adaptive immunity, wound healing, and angiogenesis and eliminating oxidative stress.

**Conclusion:**

The information obtained from bone cells and OLCS exosomes will help us discover new features of bone ageing.

**Electronic supplementary material:**

The online version of this article (10.1186/s13018-019-1163-4) contains supplementary material, which is available to authorized users.

## Background

Osteocytes, the most numerous cell populations in bone, are responsible for sensing mechanical force and acting as the “hub” of the regulatory network for bone remodelling. The osteocyte network and the lacunar canaliculae constitute a functional syncytium—the osteocytic lacunar-canalicular system (OLCS) [1–3], underscoring that the function of osteocytes cannot be implemented without cell-to-cell connections and their 3D physiological environment.

Mechanical stress is an effective stimulus for osteocytes to release messengers such as ATP, nitric oxide (NO), and prostaglandin E2 (PGE2) [4–6], which then transport through the lacunar-canalicular system (LCS) and gap junctions between adjacent cells to the osteogenic surface to prompt osteoblast-mediated bone formation and directly inhibit osteoclast activation [7–9]. Additionally, since osteocytes account for more than 90% of bone cells [10], the large number of processes in the osteocyte network possesses an immense interface for the exchange of information and substances between cells and the extracellular environment [11, 12]. Thus, steroids, hormones, and cytokines in the environment can affect the regulatory function of osteocytes by binding to their specific receptors, for example, the parathyroid hormone (PTH) [13], fibroblast growth factors (FGFs) [14], and vitamin D3 [15].

Osteocytes are also responsible for the secretion of the bone. Recently, the endocrine function of the bone has gradually gained more attention, as bone-derived hormones and substances (OCN, FGF23, and LCN2) have been identified as regulating the homeostasis of metabolism, calcium phosphorus, and even innate immune responses and cognition [16–20]. Abnormal serum levels of these known hormones and substances are considered risk factors for several degenerative diseases, including cardiovascular disease (CVD) [21, 22], chronic kidney disease (CKD) [23–25], type 2 diabetes [26], and obesity [27–29]. Furthermore, osteoimmunology studies have shown cross-regulatory mechanisms between bone and the immune system, especially in skeleton degenerative diseases such as osteoporosis and osteoarthritis. The above evidence prompts us to probe the correlation between bone secretion and ageing.

Since ageing is a time-dependent process, and osteocytes are the longest-lived cells in bone, we hypothesized that ageing should have great influence on gene transcription and cell secretion of osteocytes. Herein, by combining transcriptome-wide and proteome-wide descriptions of functional changes, the importance of osteocyte/bone cell senescence during bone ageing is emphasized.

## Methods

### Animals

SPF C57BL/6 mice and SD rats were obtained from the medical laboratory animal center of Tongji Medical University, Shanghai, China. Before experiments, the rodents were acclimatized to laboratory conditions for a week, with a commercial standard cube diet (Xietong Medical and Biological Engineering Co. Ltd., Jiangsu) and water ad libitum. The animal experiment ethics committee of Tongji University approved the animal experiments (number: TJLAC-017-015). All methods were carried out in accordance with the approved guidelines of Tongji University for Molecular Science.

### Morphological analysis and observations

Three-month-old and 20-month-old C57BL/6 female mice were sacrificed by cervical dislocation. For micro-CT analysis, the femurs (six samples in each group) were scanned by μCT50 (Scanco, Switzerland), 10 μm per slice. The parameters included the bone volume to total volume ratio (BV/TV), trabecular number (Tb.N) and cortical bone thickness (CBT). For histological analysis, 4-μm-thick paraffin sections of specimens were prepared. First, the sections were stained with hematoxylin and eosin. For observation of bone matrix, the sections were stained with Toluidine Blue, Sirius Red (Sigma, USA), and Masson Trichrome (IHC World, USA) as per the manufacturer’s instructions. For transmission electron microscopy (TEM), observations were conducted using H-7650 transmission electron microscope (Hitachi, Japan).

### Sample preparation

For RNA sample preparation, three 3-month-old (samples Y1, Y2, and Y3, weighing from 25 to 27 g) and three 20-month-old (samples O1, O2, and O3, weighing from 32 to 37 g) female C57BL/6 mice were anaesthetized by intraperitoneal infusion with 5% sodium pentobarbital. Then, the animals were perfused through the left ventricle at a constant flow of 20 ml/min with ice-cold physiological saline (PBS) for 60 s. Additionally, 3-mm segments of the cortical bone in the mid-shaft of the femur and tibia diaphysis were isolated and sampled. The bone marrow was first washed away with ice-cold PBS. To remove the cell components on the surface, the cortical bone was cut into halves lengthwise, the inside and outside surfaces were vigorously flushed with PBS, and then with ice-cold TRIzol® reagent (Ambion, USA) twice. The cortical bone segments derived from a single mouse were then smashed in TRIzol reagent, and the total RNA was extracted per the manufacturer's instructions. For exosome sample preparation, two 3-month-old (samples young1 and young2, weighing 305 and 312 g) and two 20-month-old (samples old1 and old2, weighing 399 and 376 g) female SD rats were anaesthetized by intraperitoneal infusion with 5% sodium pentobarbital. The heart was perfused with 200 ml of PBS through the left ventricle before sacrifice. The long bones were isolated under sterile conditions. The soft tissue was carefully removed, and the mid-shaft cortical bone derived from the femur and tibia was sampled. Bone marrow was removed by flushing. Then, the cortical bone was incubated in 0.25% trypsin at 37 °C for 10 min to remove the cellular components on the bone surface. The bone segments were cut into 2 × 2-mm pieces and incubated in 0.1% type I collagenase at 37 °C for 20 min. After digestion, bone segments were washed with PBS five times and centrifuged at 1000 rpm for 10 min. The precipitates were incubated in serum-free MEM at 37 °C and 5% CO_2_. After incubation for 48 h, the medium was collected and centrifuged at low speed (300 g, 10 min; 2000 g, 30 min) and ultra-high speed (10,000 g, 30 min; 150,000 g, 2 h at 4 °C) using a SW28 rotor (Beckman Coulter, USA) for exosome isolation. Two replicates were performed for each group.

### RNA sequencing

Sequencing libraries were generated using the NEBNext® UltraTM RNA Library Prep Kit for Illumina® (NEB, USA) following the manufacturer’s recommendations, and library quality was assessed on the Agilent Bioanalyzer 2100 system. Clustering of the index-coded samples was performed on a cBot Cluster Generation System using the TruSeq PE Cluster Kit v3-cBot-HS (Illumina) according to the manufacturer’s instructions. The library preparations were sequenced on an Illumina HiSeq 2000/2500 platform, and 100-bp/50-bp single-end reads were generated. Raw data in fastq format were first processed through in-house Perl scripts. Clean data were obtained by removing reads containing adapters, reads containing poly-N, and low-quality reads from the raw data. At the same time, the Q20, Q30, and GC content of the clean data were calculated. All downstream analyses were based on high-quality clean data. Bowtie v0.12.9 was used to align the single-end clean reads to UniGene sequences. HTSeq v0.6.1 was used to count the read numbers mapped to each gene. The reads per kilobase transcriptome per million reads (RPKM) of each gene was calculated based on the length of the gene and the read count mapped to the gene.

### Exosome identification

The morphology of OLCS exosomes was observed by transmission electron microscopy (Hitachi, Japan). Laser scattering microscopy (ZetaView®, Germany) was used to measure the particle size of the exosomes (Fig. 6b). The expression of exosome markers (CD9 and CD81) was confirmed by Western blotting (Fig. 6c). Anti-CD9 and CD81 antibodies were purchased from Abcam (Cambridge, UK).

### Liquid chromatography-mass spectrometry analysis

Exosomes were lysed in SDT buffer and ultrasonicated, and protein concentrations were subjected to SDS-PAGE. Proteins were then digested using the FASP protocol [30]. Two micrograms of enzymatic hydrolysis lysate (obtained by filter-aided sample preparation) were analysed using 150 μm × 20 mm Thermo EASY column SC001 traps (Thermo, USA). Liquid chromatography-mass spectrometry (LC-MS/MS) was performed using the QExactive platform in positive ion mode with a scanning range of 300-1800 m/z. Twenty fragment spectra (MS2 scan) were collected after each full scan. The resolution for MS1 and MS2 at m/z 200 was set to 70000 and 17500, respectively. The raw data were analysed using MaxQuant software (1.3.0.5). Label-free quantitation (LFQ) analysis [31] was performed, and the proteins with a certain LFQ intensity and that overlapped between two runs were selected and processed for statistical analysis using Perseus software (1.3.0.4).

### Bioinformatic analysis

Differential expression analysis was performed using the DESeq R package (1.10.1). The *P* values were adjusted using Benjamini and Hochberg’s approach. Genes with an adjusted *P* value less than 0.05 found by DESeq were considered to be differentially expressed. For the LC-MS/MS analysis, proteins with an LFQ intensity ratio between the two groups (old and young) larger than 2 and overlapped between two replicants were selected for Gene Ontology (GO) and Kyoto Encyclopedia of Genes and Genomes (KEGG) enrichment analysis performed by the clusterProfiler R package in Bioconductor [32]. GO terms and KEGG pathways with corrected *P* values less than 0.05 were considered significantly enriched.

### Statistical analyses

All statistical analyses were performed with SPSS software, version 20.0. When data sets adhered to a normal distribution, Student’s *t* test was used to evaluate the statistical differences between the two groups. The Mann-Whitney *U* test was used for data that were not normally distributed. *P* < 0.05 was considered significant. All numerical data are expressed as the mean ± s.d.

## Results

### The morphological changes in aged bone

**Fig. 1:**
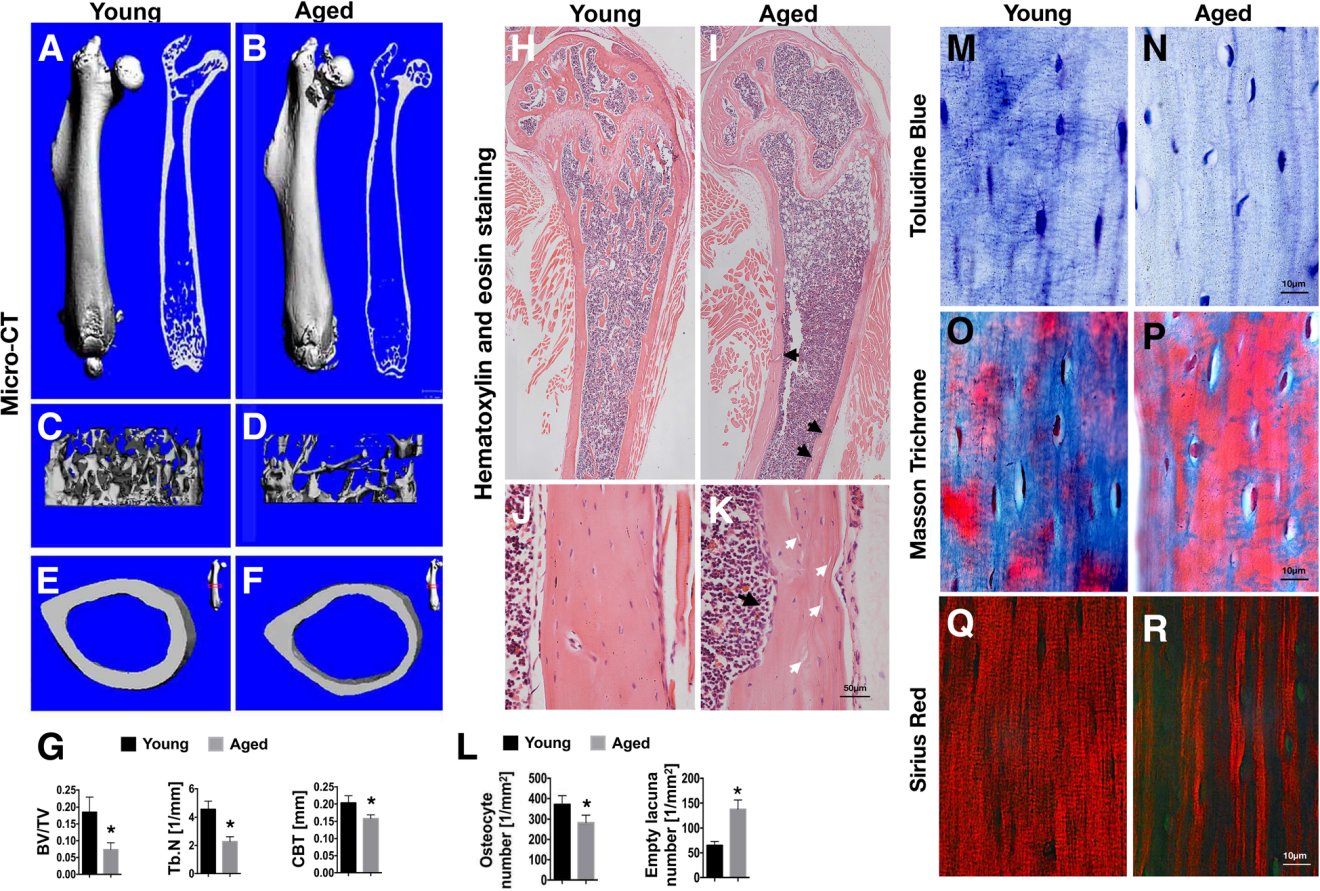
Ageing-related changes of bone and bone matrix. **a**–**f** Representative micro CT images: overall views (**a**, **b**), trabecular views (**c**, **d**), and cross section views (**e**, **f**) of young and aged mouse femurs. **g** Micro CT measurements for bone volume to total volume ratio (BV/TV), trabecular number (Tb.N), and cortical bone thickness (CBT) in mouse femurs. **h**–**k** Representative images of H&E staining of mouse femurs: low magnification (**h**, **i**) and high magnification of cortical bone area (**j**, **k**), young and aged. White arrows (**k**) indicate the empty lacunae, and black arrows indicate the resorption pits (**i**, **k**). **l** The osteocyte and empty lacuna counts per unit area in the young and aged cortical bone. **m**, **n** Representative images of Toluidine Blue staining of cortical bone, young and aged. **o**–**r** Distribution of collagen in the bone indicated by Masson’s trichrome (**o**, **p**) and Sirius Red staining (**q**, **r**), young and aged. Data in all bar plots are shown as means ± SD, *P < 0.05

**Fig. 2:**
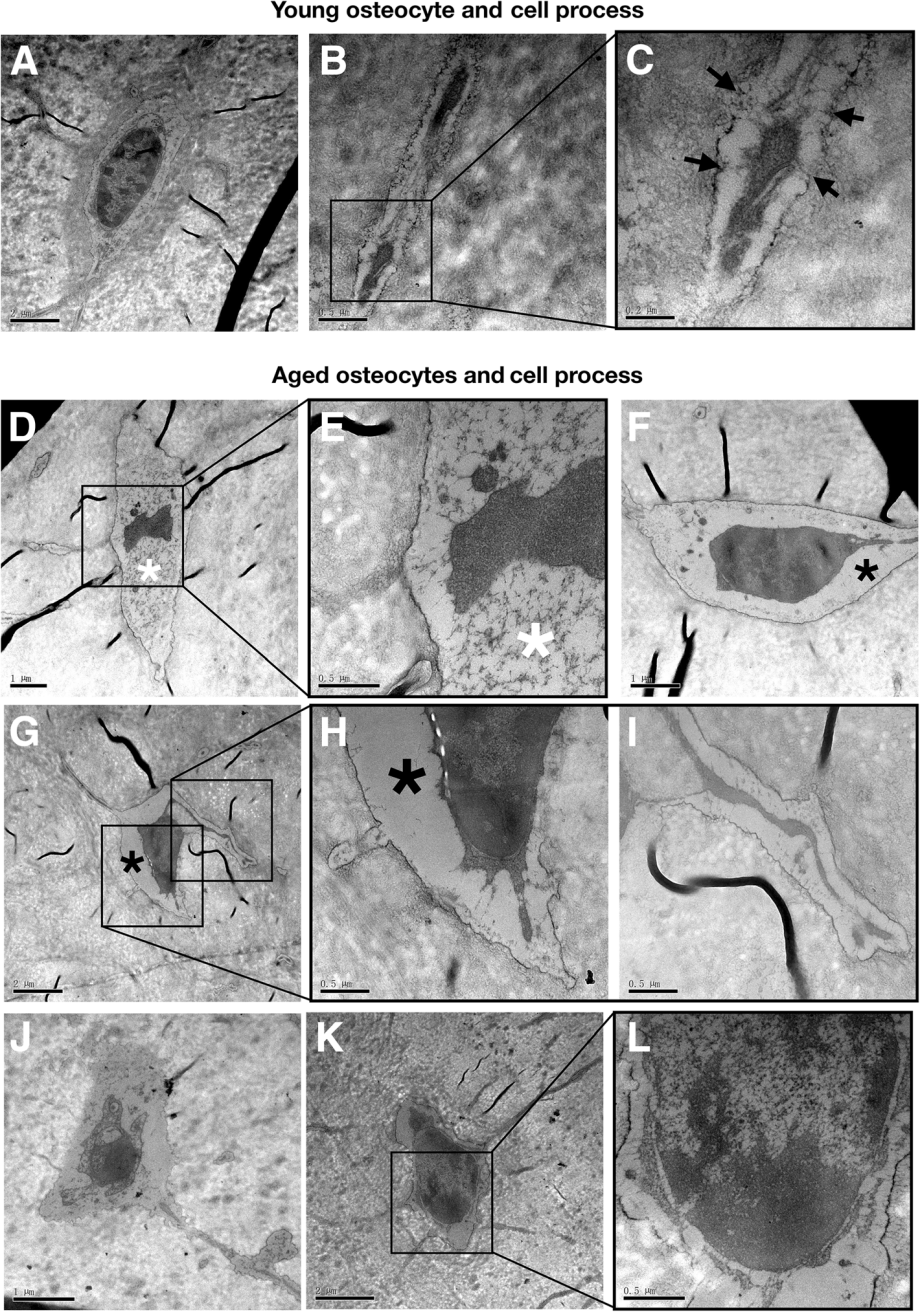
Ageing-related morphological changes in osteocytes. **a**–**c** Micromorphology of osteocytes in young mouse cortical bone, shown by TEM. Osteocyte shape (**a**) and the ultrastructure of osteocyte processes (**b**, **c**). The connecting fibers between cell processes and pericanalicular matrix, indicated by a black arrow (**c**). In the aged bone, osteocyte dendrites lack fibers anchoring to the canalicular wall (**i**). **d**–**l** TEM images of osteocytes in aged mouse cortical bone. Aged osteocytes show a variety of abnormal cytomorphological features: cytoplasmic lysis (**d**, **e**), indicated by a white star; empty cytoplasm (**f**–**h**), indicated by a black star; and (**j**–**l**) abnormal forms of the nucleus, shrinkage (**j**), and swelling (**k**, **l**)

Compared with young bones, aged bones displayed significant decreases in bone mass indicated by the reduced micro-CT parameters of BV/TV, Tb.N, and CBT (Fig. 1a–g). Histological analysis showed a decreased number of osteocytes and an increased number of empty lacunae in aged relative to young cortical bone (Fig. 1l). Compared with young bone, the inner surface of aged cortical bone was less smooth with more visible resorption pits, below which empty lacunae were easily observed (Fig. 1h–k). To further investigate the condition of the extracellular matrix, Toluidine Blue, Masson, and Sirius Red staining were performed. The results showed abundant dendrites connecting into a network around young osteocytes in young bone (Fig. 1m), while continuous dendrites were rare in aged cortical bone (Fig. 1n). Masson staining revealed predominantly blue staining in young cortical bone (Fig. 1o); in aged bone, however, bone matrix was mainly stained in red (Fig. 1p), indicating a change in collagen composition in bone matrix during ageing. Sirius Red staining demonstrated poor densification and loose tissue structure of collagen fibres in aged bone matrix (Fig. 1q, r). Further, morphological abnormalities in the shapes of cell bodies, dendrites, and nuclei were shown by TEM (Fig. 2). The osteocytes of aged mice showed many morphological abnormalities, such as cytoplasmic lysis, empty cytoplasm, and abnormal nuclei (Fig 2d–l). Moreover, tethering elements between osteocyte dendrites and the canalicular wall were visible in young bone (Fig. 2c) but were absent in aged bone (Fig. 2i).

### GO and KEGG pathway enrichment analysis of the DEGs between aged and young cortical bone cells

**Fig. 3:**
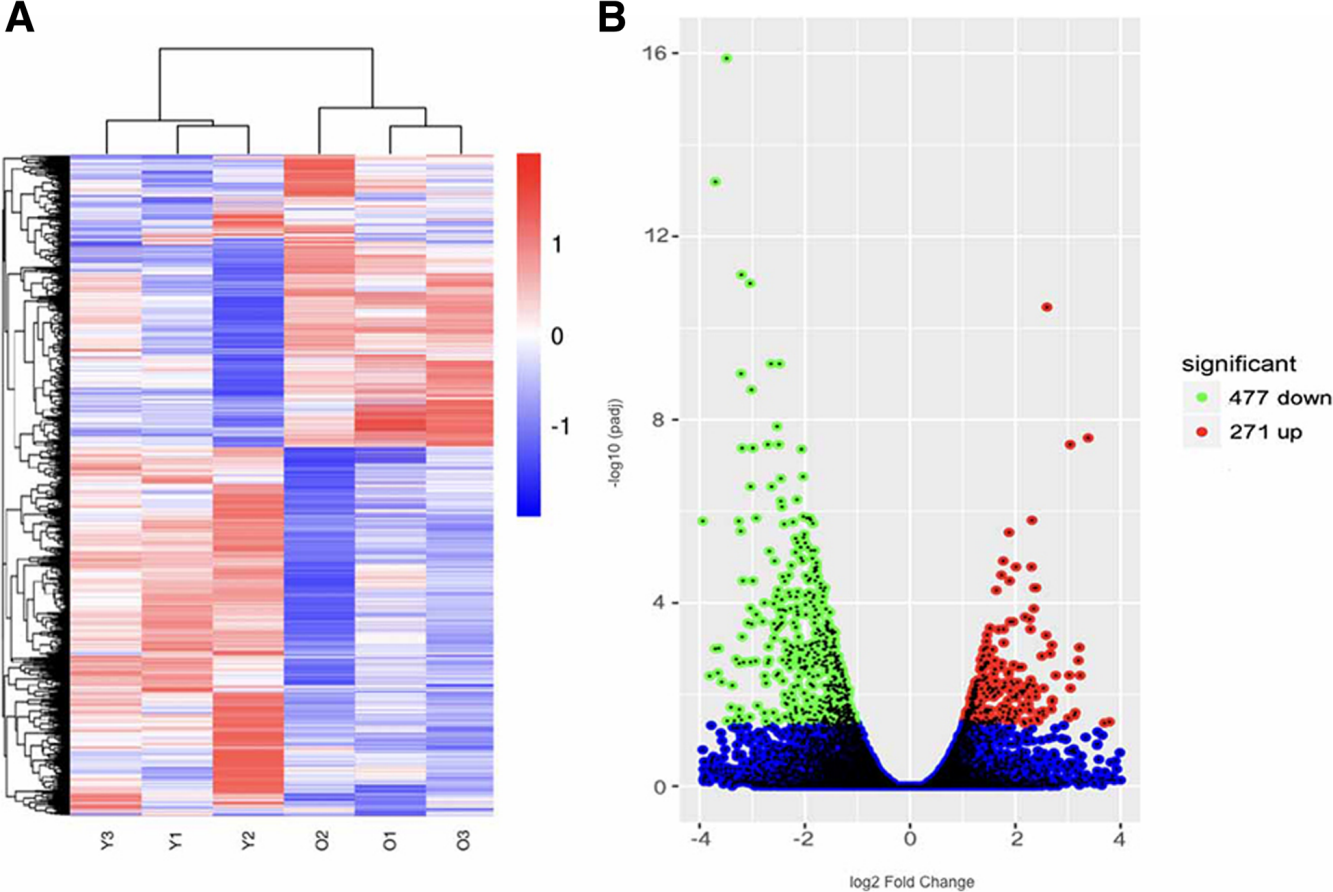
Transcriptome analysis of aged and young mouse cortical bone cells by RNA-seq. **a** Heat map of differential gene expression between the young (Y) and aged (O) groups. **b** Volcano plots for the differentially expressed genes (DEGs), aged vs young. Numbers are given for the upregulated (red plots) and downregulated (green plots) DEGs

**Fig. 4:**
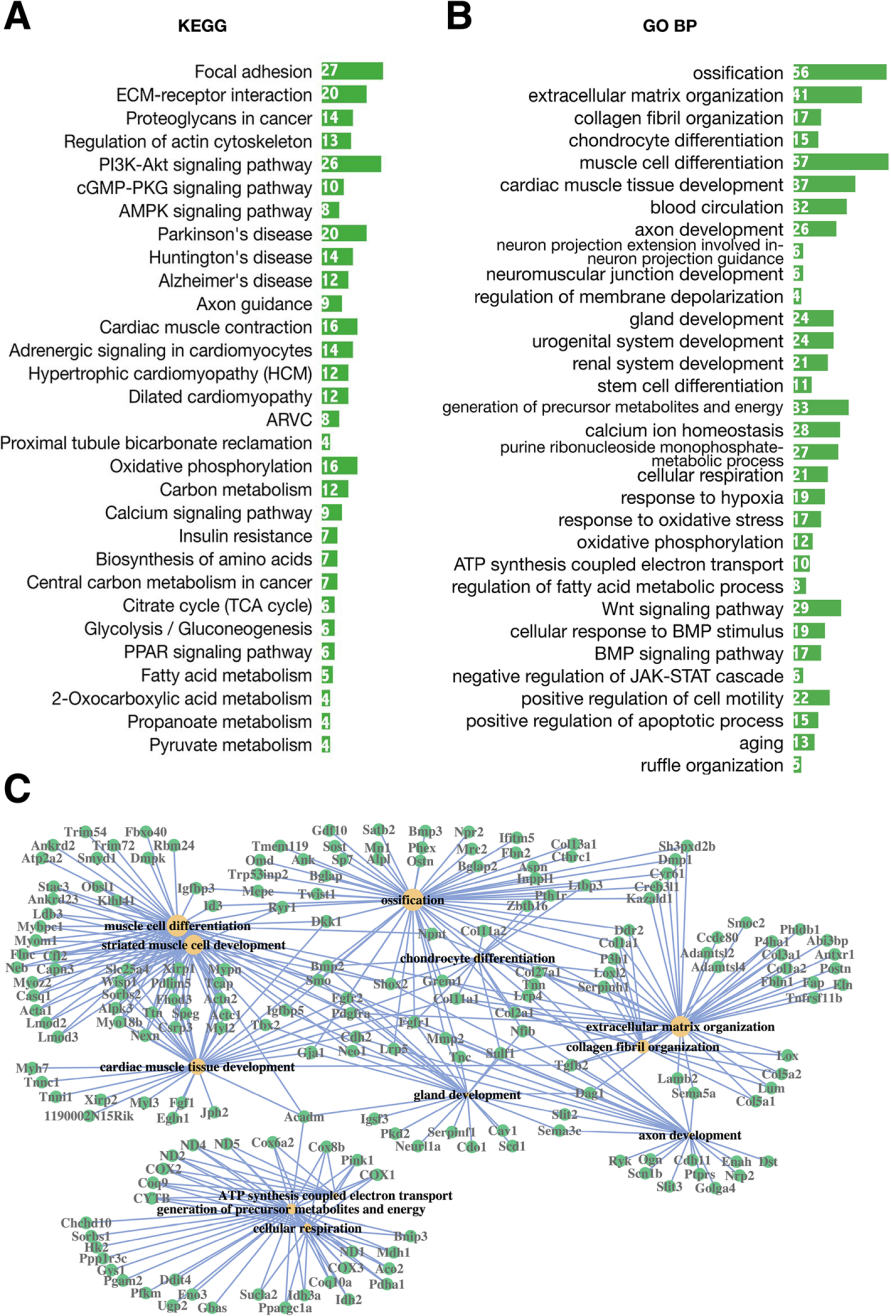
Top GO terms and KEGG pathways among downregulated DEGs. Enriched KEGG pathways (**a**) and GO terms (**b**) for downregulated DEGs, aged vs young. The abscissa represents DEG numbers. **c** Network of the most enriched GO terms (BP) and associated DEGs, downregulated, aged vs young. The sizes of the yellow nodes are proportional to the numbers of DEGs related to a given GO term

**Fig. 5:**
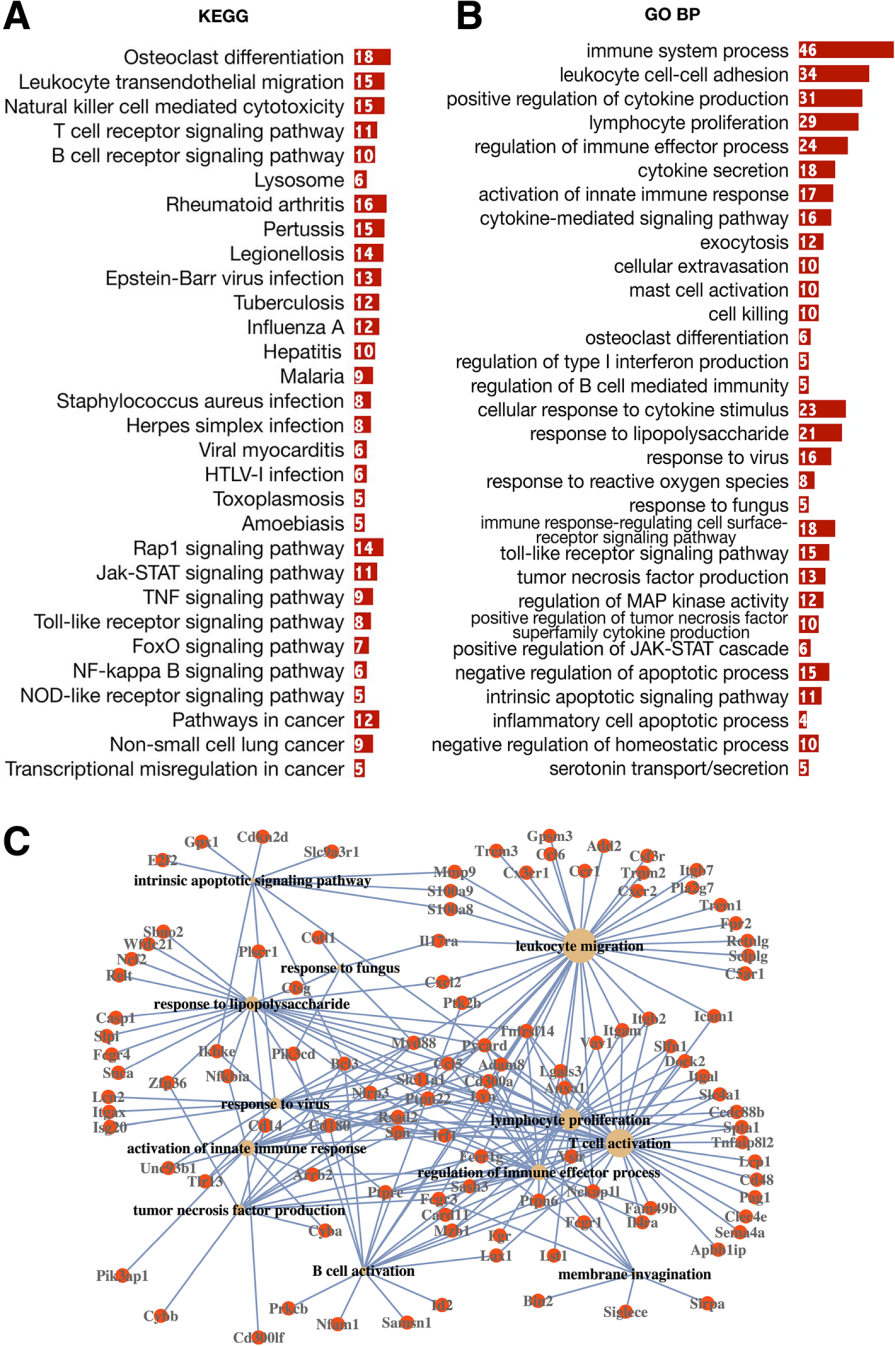
Top GO terms and KEGG pathways among upregulated DEGs. Enriched KEGG pathways (**a**) and GO terms (**b**) for upregulated DEGs, aged vs young. The abscissa represents DEG numbers. **c** Network of the most enriched GO terms (BP) and associated DEGs, upregulated, aged vs young. The sizes of the yellow nodes are proportional to the numbers of DEGs related to a given GO term

Differential expression analysis of the RNA-seq results identified 271 upregulated and 477 downregulated differentially expressed genes (DEGs) between aged and young cortical bone cells (Fig. 3). The most enriched GO terms and KEGG pathways are listed in Figs. 4 and 5. With reference to the given functions of osteocytes, the key molecules in our DEGs that can perform and represent these functions are highlighted, as changes in the expression levels of these functional molecules can partially reflect the characteristics of bone cell senescence.

### Exosome identification and exosomal proteins identified by LC-MS/MS

Given that secretory substances of the bone can regulate the functions of adjacent bone cells (OCs, OBs, MSCs) or even distant organs, exosomes, small membrane-bound vesicles that carry biological macromolecules to target sites, might be an effective mediator of bone secretion and could show functional alterations from the perspective of bone secretion. In this context, OLCS exosomal proteins of both groups were isolated for LC-MS/MS analysis.

**Fig. 6:**
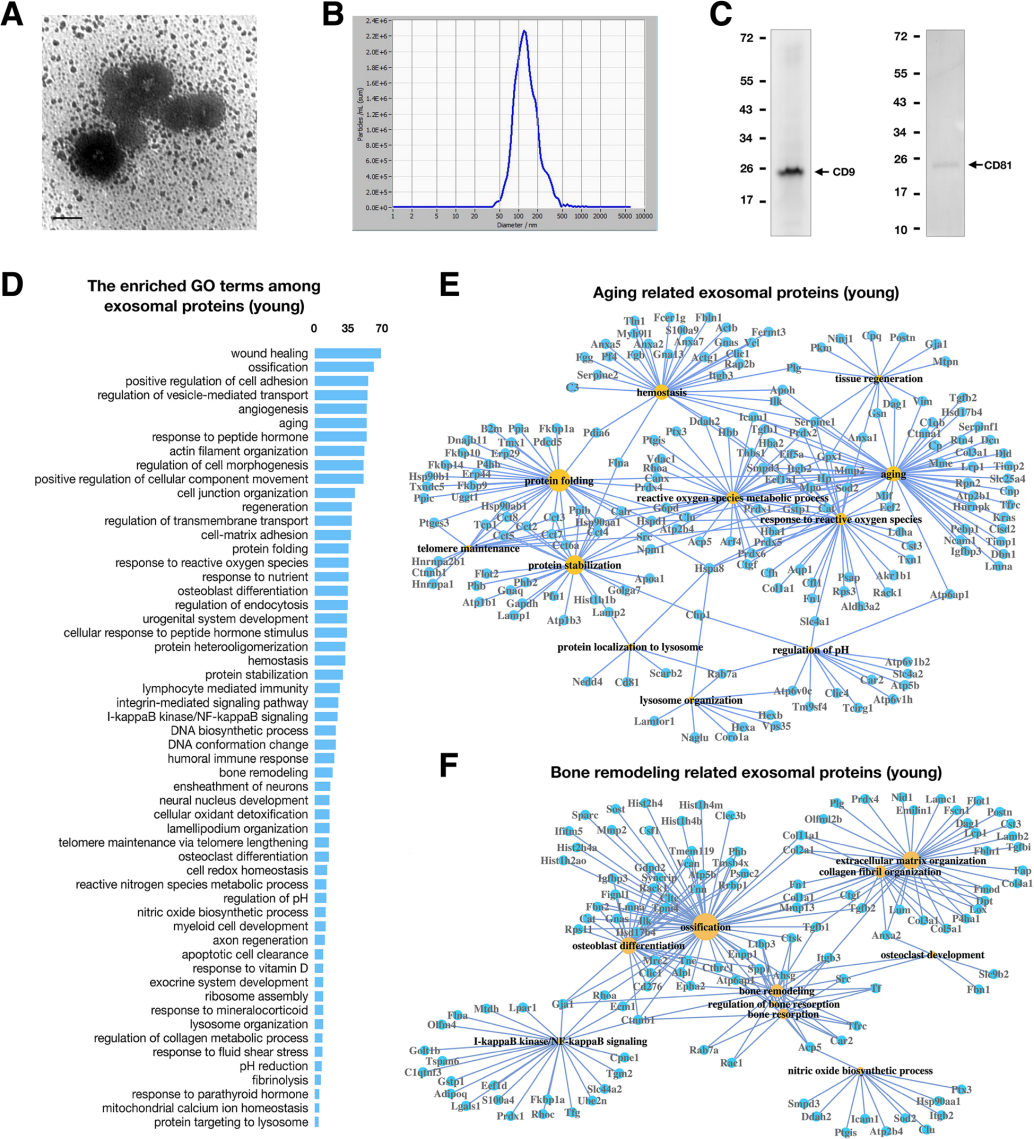
Exosome characterization and proteomics analysis of exosomal proteins. (**a**) Representative TEM image of exosomes isolated from young rat cortical bone (scale bar = 100 nm). **b** Particle size measurement of exosomes by dynamic light scattering. **c** Western blot analysis of CD9 and CD81 in exosomes. **d** The most enriched GO terms among the proteins identified in young rat OLCS exosomes. The abscissa represents protein numbers. **e** Network of the ageing-related GO terms and associated proteins identified in young rat OLCS exosomes. **f** Network of the bone remodelling-related GO terms and associated proteins identified in young rat OLCS exosomes. The sizes of the yellow nodes are proportional to the numbers of DEGs related to a given GO term

Three conventional experiments were performed to confirm the exosomal nature of isolated vesicles: visualization of the isolated vesicles by electron microscopy revealed the presence of spherical structures approximately 100 nm in size (Fig. 6a); particle size measurement indicated a size range between 80 and 200 nm, with a peak size of 120.9 nm in diameter (Fig. 6b), and the expression of the general exosome markers CD9 and CD81 was validated by Western blotting showing MWs of 25 and 20 kDa, respectively (Fig. 6c).

Further, in subsequent LC-MS/MS analysis, a total of 1019 proteins in young and 700 proteins in aged OLCS exosomes were identified. A large number of exosome-associated proteins were found as evidence that authentic exosomes were obtained: generally, more than 58% (592 out of 1019 in the young group) and 63% (444 out of 700 in the aged group) of the identified proteins were enriched in the GO cellular component term “extracellular exosomes” (data not shown), which was at the top of the list sorted by *P* value, and the following GO terms: focal adhesion, membrane, vesicles, extracellular matrix, and membrane raft (data not shown), which are all exosome-related cellular components. Furthermore, many exosome markers were found both in young and aged OLCS exosomes, including the commonly studied exosome markers tetraspanins (CD9, CD63, CD81), flotillin and caveolin (FLOT1, FLOT2, CAV1), major histocompatibility complex protein (RT1.Alu, RT1-CE14, RT1-Bu alpha, RT1-Bu beta, RT1.Alu, RT1.Ab), and integrins (ITGA1, 2, 5 and ITGB1, 2, 3, 6). Moreover, many other potential markers were also included: annexins (ANXA1, 2, 3, 4, 5, 6, 7, and 11), transcription factors (EF1A and EF2), heat shock proteins (HSPA8, HSP90AA1, HSP90AB1, and HSPD1), phosphatidylserine-binding protein (MFGE8/lactadherin), and growth factor receptor (EGFR [only in the young group]). Last but not least, several proteins that are considered to be absent or underrepresented in exosomes have not been identified, for example, Argonaute/RISC complex (AGO) and golgin (GM130).

### GO analysis of DEPs in exosomes derived from young and aged cortical bone

**Fig. 7:**
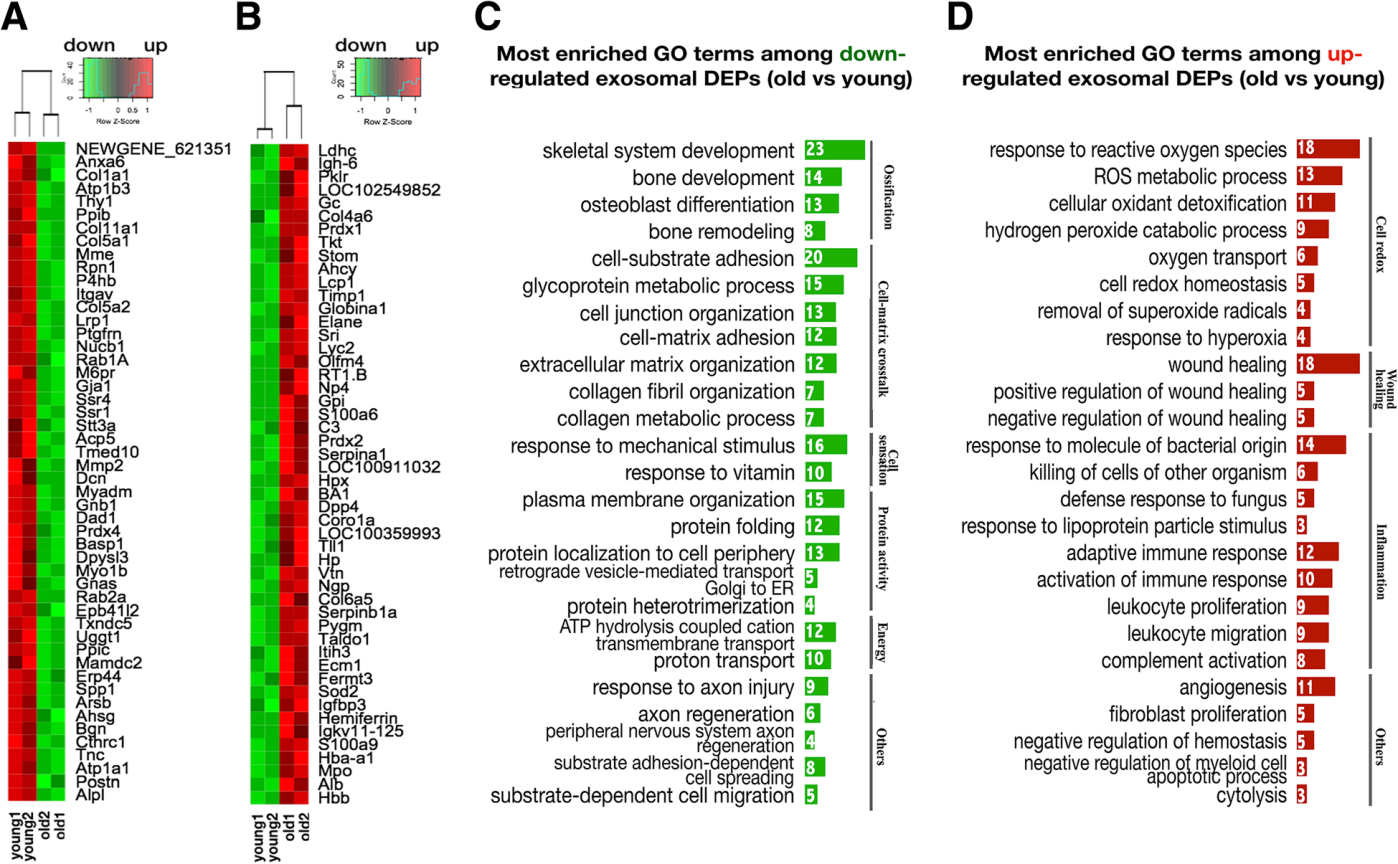
GO analysis of the differentially expressed exosomal proteins. **a**, **b** Heatmap depiction of the top 50 overlapping downregulated (**a**, green) and upregulated (B, red) proteins in OLCS exosomes, aged vs young. **c**, **d** The most enriched GO terms among downregulated (**c**) and upregulated (**d**) exosomal proteins, aged vs young. The numbers are DEP counts for each GO term

**Fig. 8:**
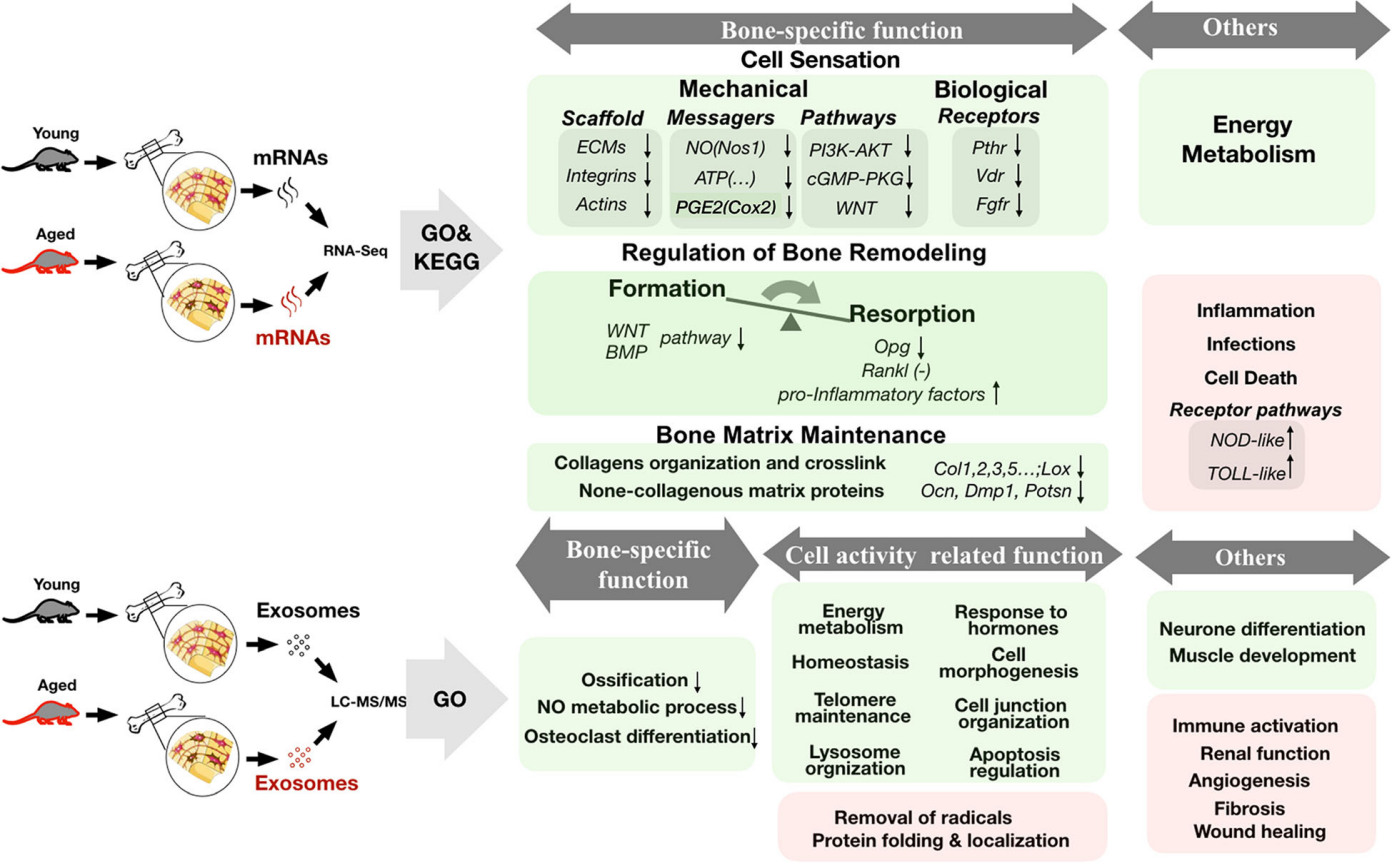
Ageing characteristics of bone indicated by transcriptomic and exosomal proteomic analysis of cortical bone cells. The scheme enumerates the main findings of this study. Up-and down-regulations of bone cell functions and signaling pathways are shown in pink and light green columns, respectively

The top GO terms (BP) for the young group (with an input of 1019 proteins) are listed in Fig. 6d. Among them, the ageing- and bone remodelling-related GO terms and associated proteins are displayed in Figs. 6 e and f, respectively. The differing GO terms between the aged and young groups (the same GO terms between two groups were eliminated) were preserved and are listed in Table 1. LFQ analysis identified 236 downregulated and 177 upregulated differentially expressed proteins (DEPs) in OLCS exosomes of the aged group relative to that of the young control. The top 50 DEPs (sorted by log2 fold change in LFQ intensity) are listed by heatmap depiction in Figs. 7 a and b. The enriched GO terms among downregulated and upregulated DEPs are listed in Figs. 7c and d. Ageing characteristics of bone indicated by the results of this study were summarized in Fig. 8.

**Table 1:** The most enriched GO terms (BP) among proteins identified in exosomes of both groups

	GO terms among proteins identified in exosomes (young)	GO terms among proteins identified in exosomes (aged)
Protein	Establishment of protein localization to membrane (35) Protein peptidyl-prolyl isomerization (10) Chaperone-mediated protein complex assembly (4) Positive regulation of protein secretion (24)	Protein depolymerization (11) Cellular protein complex disassembly (13) Positive regulation of protein localization to nucleus (14) Protein nitrosylation (4)
DNA	DNA conformation change (23/217) Telomere maintenance (15/113)	None
Cell appendage morphogenesis	Lamellipodium organization (16) Ruffle organization (10) Membrane raft assembly (4) Podosome assembly (6) Regulation of cell junction assembly (12)	None
Metabolism	Nitric oxide metabolic process (12) Regulation of nitric oxide biosynthetic process (12) Tricarboxylic acid metabolic process (8)	None
Cellular transportation	Golgi vesicle transport (25) Energy coupled proton transmembrane transport (12) Positive regulation of protein transport(45)	None
Cell clearance	Lysosome organization (5) Protein targeting to lysosome (5)	None
Cell homeostasis	Regulation of pH (13)	Negative regulation of homeostatic process (18)
Cellular response	Cellular response to peptide hormone stimulus (34) Response to parathyroid hormone (6) Response to angiotensin (7) Response to mineralocorticoid (10)	Acute-phase response (10) Response to fungus (7) Response to immobilization stress (7) Response to lipopolysaccharide (34) Killing by host of symbiont cells (4)
Cell stress	Response to endoplasmic reticulum stress (23)	Respiratory burst (5) Cellular response to oxidative stress (23)
Cell death	Negative regulation of apoptotic signaling pathway (15)	Regulation of intrinsic apoptotic signaling pathway in response to DNA damage (6) Cytolysis (6) Membrane invagination (9)
Immunologic process	I-kappaB kinase/NF-kappaB signaling (24)	Activation of immune response (27) Positive regulation of leukocyte-mediated immunity (13) B cell-mediated immunity (16) Positive regulation of inflammatory response (13) Neutrophil migration (11) Granulocyte chemotaxis (12) Positive regulation of T cell activation (16) Leukocyte migration (29) Cell-cell recognition (9) Complement activation, lectin pathway (4)
Cell-matrix adhesion	Regulation of cell-matrix adhesion (16)	None
Skeletal system	Positive regulation of ossification (14) Osteoclast differentiation (5)	None
Nervous system	Axon ensheathment (17) Peripheral nervous system axon regeneration (5) Glial cell differentiation (25)	Negative regulation of nervous system development (23)
Other systems/tissue	Myeloid cell development (12) Regulation of muscle contraction (18)	Fibroblast proliferation (12) Renal system development (25) Regulation of coagulation (16) Smooth muscle cell proliferation (14)

The numbers in parentheses are the counts of proteins annotated for each GO term

## Discussion

### The morphological changes in aged bone and osteocytes

Based on the morphological evaluation and observation of young and aged long bones (Figs. 1 and 2), the main phenotypes of aged cortical bone can be generally summarized as (1) loss of bone mass, (2) changes in bone matrix (structure and composition), and (3) poor osteocyte status. We assume that poor osteocyte status may be the root cause of bone ageing phenotypes, and molecular evidence that can link the senescence of osteocyte network to bone ageing urgently needs clarification.

### Acquisition of biological information from bone cells and its limitation

In this study, the biological information was obtained directly from the cortical bone. So, the key issue is ensuring that osteocytes are the most abundant cells in the sampling site. In this context, we took a small segment of cortical bone where the osteocytes showed highly differentiated morphology with abundant dendrite connectivity. Heart perfusion can effectively remove the blood cells from bone tissue. The pretreatment of the cortical bone surface by a quick flush with TRIzol reagent before RNA extraction and by a short digestion with trypsin and collagenase before exosome extraction can reduce the influence of adherent cells, including osteoblasts (OBs), lining cells, and osteoclasts (OCs). Nevertheless, it is difficult to remove the vascular endothelial cells (VECs) in the bone matrix. Research on the quantitative morphometry of the bone vasculature has shown that the vascular volume density accounts for 00.13% of the cortical total volume in the region of the murine femoral mid-diaphysis [33]. Additionally, histological observations of serial sections in our preliminary experiment suggested that the proportion of osteocytes was more than 90% (90.49%) of the total number of cells in the middle 3-mm segments of the cortical bone (Additional file 1: Figure S1 and S2). Although the osteocytes are the majority of cells in our sampling site, the information we obtained was not from pure osteocytes but a mixture, which we consider as the limitation of this study. More accurate sampling methods and cutting-edge sequencing methods, such as laser microdissection based on hard tissue and single-cell sequencing, may be helpful to optimize the methods of this study.

### DEGs involved in bone mechanosensation

The expression of many key factors involved in the bone mechanosensation is downregulated in the aged cortical bone. First, the critical structural molecules that allow osteocytes to bear mechanical force are downregulated (Fig. 4): integrins and related factors (e.g., Itga10, Itgb4, Chad, Fap), components of focal adhesions (e.g., Lamb2, Thbs2/4, Tnc, Tnn, Pdgfra), cytoskeleton proteins (e.g., Actn2, Cav1, Flnc, Myl2, Mylk4), and the main calnexin (Cx43/Gja1) that composes the gap junction between osteocytes. Second, key genes whose products involve in mechanical stimulation induced second messenger production: key enzymes involved in NO, PGE2, and ATP synthesis (e.g., NOS1, COX1-3, ND1-5), factors involved in the regulation of homeostasis and the intracellular flow of calcium ions (Casq1, Jph2, Trdn, Jsrp1, Hrc, Capn3, Ryr1), and Sost, an osteocyte-specific marker that integrates osteocyte mechanotransduction and bone mass by antagonizing Wnt/beta-catenin signalling [34, 35]. Additionally, downstream signalling elicited by mechanical force-induced messengers, such as cGMP-PKG, PI3K-AKT, and WNT signalling, is downregulated (Fig. 4a). Taken together, these lines of evidence tend to show the attenuation of the mechanical sensing of aged bone cells.

### Hormone receptors

Here, the gene expression of some hormone receptors, including Pth1r, VDR, Fgf1r, and Fgf2r, was downregulated in aged cortical bone. Parathyroid hormone (PTH), secreted by the parathyroid glands, plays a central role in maintaining bone metabolic homeostasis by binding to the PTHR, a G protein-coupled membrane-spanning receptor. Activation of PTH1R increases both bone formation and resorption by inhibiting Sost expression and elevating the RANKL/OPG ratio, respectively [13]. Vitamin D binds to vitamin D receptor (VDR), as is the case with nuclear receptors. Its main function is to mediate calcium and phosphorus metabolism by regulating the expression of the molecules (e.g., DMP1, MEPE, PHEX) involved in the synthesis and secretion of inorganic pyrophosphate and the regulation of bone matrix mineralization [15]. The expression of FGF23, the core regulator of body calcium and phosphorus [14], has been demonstrated to be influenced by both PTH/PTHR and vitamin D/VDR signalling through the fibroblast growth factor receptor (FGFR) [13, 36]. Given their important role in mediating the calcium phosphate homeostasis between the bone and the circulatory system, downregulation of these receptors may alter how cells perceive and respond to hormones during ageing.

### DEGs involved in bone formation and resorption

Many key osteogenic molecules and pathways are downregulated in aged group, including members of the BMP and WNT signalling pathways (Tgfb2, Bmp2, Wnt5b, Wnt16, Lrp4, Lrp5) and their target genes (Gremlin, Twist, Ocn, Postn, Gja1, Opg, Cdh2/11/15, and Alp1) (Fig. 4b). Moreover, many regulators of these two pathways are also downregulated; some are positive regulators, e.g., Col1a1, Bambi, Cav1, Fgfr2, and Kank1, while some are inhibitors, including Twist, Dkk1, Nbl1, and Cdh2. Downregulation of these regulatory factors indicates that the dominant role of bone cells in regulating bone formation weakens during ageing.

Regarding the formation and maintenance of bone matrix, gene expression levels are downregulated for a variety of collagens (e.g., COL1, COL2, COL3, COL5, COL8, COL11), molecules involved in collagen fibril organization (e.g., LUM, P3H1, GREM1, LOX), and many non-collagenous bone matrix proteins (OCN, ON, POSTN, DMP1, MEPE, PHEX) (Fig. 4). The lack of these molecules could affect the mineralization and mechanical properties of the extracellular matrix.

It has been demonstrated that the mediation of osteoclastic bone resorption depends mainly on the receptor activator of nuclear factor kappa-Β ligand (RANKL)/osteoprotegerin (OPG) ratio. RANKL, produced by osteocytes, is the major osteoclast differentiation factor [37, 38]. Although our data showed no significant difference in RANKL expression, downregulation of Opg/Tnfrsf11b may increase the RANKL/OPG ratio, which could be the reason for the excessive bone resorption in the aged bone. In addition, the upregulation of several key genes of osteoclast stimulating factors, such as Adam8, Tyrobp, Sbno2, and Ccr1, could promote osteoclast formation. Furthermore, higher expression levels of genes involved in regulating proinflammatory cytokine responses are positively associated with osteoclast activation in the aged bone.

### DEGs related to immunity

From our data, it is obvious that the proinflammatory state is the most significant feature of bone aging, as the upregulated genes were enriched in many inflammatory GO terms involved in both innate and adaptive immunity. In innate immunity, pattern recognition receptors (PRRs), a group of receptors that activate the formation of inflammasomes by identifying pathogen-associated molecular patterns (PAMPs) and damage-associated molecular patterns (DAMPs) in the environment, play important roles in regulating pathogen recognition and activation of the intracellular inflammatory response [39]. In our data, pattern recognition-related inflammatory molecules and pathways were upregulated in the aged group (Fig. 5), including (1) two important pattern recognition receptor (PRR) pathways—the Toll-like receptor and NOD-like receptor signalling pathways (Fig. 5a); (2) molecules and signalling pathways downstream of PRRs that trigger innate immunity, including NF-κB signalling (Fig. 5a) and genes related to interferon production (Irf1, Ptpn22, Myd88, Cd14, Pycard); (3) NLRP inflammasome components (Nlrp3, Pycard, Card11); and (4) molecules that are regulated in response to viruses, fungi, and lipopolysaccharide (Fig. 5a). These lines of evidence suggest that pathogen infections may be the cause of the enhanced proinflammatory state in aged cortical bone. In addition, a large number of upregulated DEGs were enriched in GO terms involved in adaptive immunity, such as regulation of cytokine production and secretion, tumour necrosis factor (TNF) production, lymphocyte proliferation, differentiation, and migration (Fig. 5b). It is worth mentioning that some known regulators of adaptive immunity were found in OLCS exosomes (see below in the “Immune-associated proteins” section), and most of them were upregulated in the aged group, which might be further evidence for the involvement of aged bone cells in the regulation of adaptive immunity.

On the other hand, there are many receptors of proinflammatory cytokines whose gene expression levels were increased in the aged group: some of them are involved in the mediation of immunosuppressive signals, such as Lilrb4, Pilra, Il10ra, and Il1r2; some play pathogenic roles in inflammatory and autoimmune diseases such as rheumatoid arthritis, for example, Il17ra; and Il18rap and Il21r, whose products are involved in the activation of signalling pathways associated with inflammation, including NF-κB, MAPK8, and JAK-STAT. Changes in the expression levels of these molecules could affect the responses of aged bone cells to inflammatory factors. Notably, molecules negatively regulating JAK-STAT signalling were downregulated (Fig. 4b), while molecules positively regulating the JAK-STAT cascade were upregulated in the aged group (Fig. 5b). Given that JAK-STAT signalling is considered to have the capacity to mediate the responses of target cells to inflammatory cytokines and plays an important role in regulating cell apoptosis [40], the active regulation of JAK-STAT signalling might be the link between cell responses to inflammatory cytokines and cell death.

### DEGs involved in cellular energy metabolism

Mitochondrial and endoplasmic reticulum (ER) insufficiencies are widely considered to be putative hallmarks of cell senescence [41]. Here, signs of decline in energy metabolism in the aged bone cells were evident: first, in the aged group, of the 69 downregulated transcripts whose products serve mitochondrial functions, many are involved in ATP production (tricarboxylic acid cycle, respiratory chain, and oxidative phosphorylation). Downregulation of these key genes may directly reduce the cellular energy supply. In addition, several downregulated molecules are key components of complex I (ND1-4) and complex IV (COX1-3) in the respiratory chain, whose dysfunction could cause electron leakage and increase the production of reactive oxygen species (ROS), one of the most important factors for cell senescence. Finally, the gene expression of several key enzymes that respond to cellular stress was downregulated: for example, LONP, which can protect cells from various stress conditions by regulating mitochondrial metabolism and repairing mitochondrial DNA [42], and PINK, which plays an irreplaceable role in mediating mitochondrial autophagy [43]. In addition, the products of some DEGs located in the ER were downregulated. FKBP9, CRTAP, and FKBP10 function as protein-folding factors mediated by chaperones [44]. MNF2 and VDAC are typical key factors on the ER–mitochondria interface [45, 46]. These changes in energy metabolism could be the root causes of bone ageing.

Changes in some cellular metabolism pathways are also important clues for determining the underlying mechanisms of bone ageing. The FoxO signalling pathway is regulated by and counteracts external changes that disturb homeostasis, including metabolic stress, oxidative stress, and growth factor deprivation [47]. According to the KEGG analysis, the FoxO signalling pathway was upregulated in the aged group, which may be further corroboration of the severe cellular stress in aged bone cells. Furthermore, FoxO signalling may have special cellular functions in the bone: it has been demonstrated that activation of FoxO in osteoblasts can hinder bone formation, as it can competitively inhibit the WNT pathway by diverting β-catenin from TCF- to FoxO-mediated transcription [48]. Moreover, the FoxO pathway is normally negatively regulated through phosphorylation mediated by the PI3K-AKT pathway in diverse organisms [49]. Meanwhile, PI3K-AKT is responsible for mechanical force-induced osteogenesis in the bone [8]. Consistently, in our data, FoxO signalling was upregulated (Fig. 5a) and accompanied by the downregulation of PI3K-AKT and WNT signalling (Fig. 4a, b). Thus, the crosstalk among these three pathways could be the molecular explanation and underlying link between the bone loss and decline in cell function that occur during ageing.

### The loss- and gain-of-function of OLCS exosomes with age indicated by GO analysis among exosomal proteins

The results revealed that the skeleton could secrete large amounts of regulatory proteins involved in regulating a wide range of cellular activities, from regulating energy metabolism and protein transportation to cell differentiation, morphogenesis, and movement (Fig. 6d). Additionally, biological processes involving the maintenance of cell homeostasis and anti-senescence were included, such as molecules involved in the regulation of telomere maintenance, protein folding and stabilization, tissue regeneration, lysosome organization, and proteins in response to ROS and hormones (Fig. 6d, e).

In clear contrast to young controls, the aged OLCS exosomes lacked functional proteins for cellular activities, including maintenance of the morphological properties of cells, maintenance of cell homeostasis, and regulation of cellular metabolism and transportation (Table 1). Moreover, the results revealed a functional decline in the regulation of DNA conformation, telomere maintenance, protein complex assembly, and localization (Table 1). In addition, proteins involved in lysosome organization and cellular responses to hormones (parathyroid, angiotensin, and mineralocorticoid) were absent in the aged group (Table 1). On the other hand, the enriched proteins in the aged group were mainly associated with activation of immune responses, cell damage, and cell death (Table 1). Most of the features mentioned above mirrored the findings at the RNA level; some of them have been demonstrated or categorized in existing studies linked to cell senescence [41] and could elucidate both the causes and consequences of bone ageing.

### Proteins identified in OLCS exosomes

#### Senescence-associated secretory phenotype

Studies have demonstrated that aged cells can secrete proinflammatory factors, growth factors, chemokines and proteases to mediate age-related inflammatory responses, wound healing, and even tumour progression; this condition is now referred to as the senescence-associated secretory phenotype (SASP) [50, 51]. Here, several classical SASP proteins, including TGF-β2, OPG, MMP9, TIMP1, MIF1, PRDXs, and IGFBP3, were found to be upregulated in aged OLCS exosomes. Among them, TGF-β2 and OPG are important mediators of bone metabolism; serum levels of TGF-β2 and OPG have shown significant positive correlations with bone turnover markers in Chinese women [52]. TIMP1, a tissue inhibitor of metalloproteinases (MMPs), is expressed in both osteocytes and osteoblasts and maintains the balance of bone matrix degradation by regulating MMP levels [53]. Moreover, TIMP/MMPs can also accommodate the immune influx in the extracellular matrix [54]. MIF1 is known to activate several transcription factors and stress kinases that can mediate inflammatory and tumourigenic signalling, e.g., NF-κB and AMPK [55]. Thus, the increased expression of these SASP proteins in senescent OLCS exosomes may explain how aged bone cells contribute to the environmental effects of adjacent and distal cells and systems.

#### Immune-associated proteins

Our analyses of the immune-associated proteins in OLCS exosomes have shown interesting findings regarding the cross-regulation between the skeleton and the immune system: First, approximately 10% (85 out of 1019 proteins identified in the young group, and 76 out of 700 proteins in the aged group) of the identified proteins found in OLCS exosomes were enriched in immune-related GO terms, and approximately 80% (67 out of 85/76 in young/aged group) of them overlapped between the young and aged groups. Although most of them were found to have a higher LFQ intensity in aged OLCS exosomes than in young ones, their overall composition in exosomes was relatively constant across age. To some extent, these results indicate that there is a certain group of molecules in OLCS exosomes, both aged and young, that may serve an immune regulatory function. Second, among these overlapping immune-associated proteins were some key factors that have been shown in existing studies to regulate the activities of both immune cells and bone cells, including CD44, CD47, CD59, TGF-β2, and GSN. Among these, CD44 is a novel marker of osteocytic differentiation in bone [56]; meanwhile, it is also widely expressed in many kinds of immune cells (T cells, granulocytes, monocytes) and is critical for their maturation [57, 58]. CD47, known as a “don’t eat me” signal to macrophages, plays a critical role in immune cell activation [59, 60], and it also has a profound effect on skeletal remodelling and bone maintenance through its actions on both osteoblasts and osteoclasts [61]. For CD59, this molecule was recently demonstrated to be a regulator of bone growth and homeostasis by interfering with the complement system in innate immunity [62]. TGF-β, a multifunctional cytokine, plays integral roles in the regulation of adaptive immunity, mainly through activating T cells and effector and regulatory T cells [63]; in the bone, it is a critical regulator coupling of bone formation and resorption [64]. All these proteins, except CD44, showed increased expression in aged OLCS exosomes. Similarly, many other immunoregulatory molecules were upregulated in aged OLCS exosomes, including several complement molecules and regulators: C3, C1QA, C1QB, C1QC, C4A, C8B, MBL1, MASP2, FCNB; positive regulators of T cell/B cell activation: SLC4A1, SPN, TFRC, ANXA1, CORO1A, FLOT, PNP, PTPRC; and regulators of leukocyte proliferation, migration, and aggregation: S100A9, S100A8, PNP, PLG, MIF, ECM1, SERPINE1, SLC4A1, GSTP1, ELANE.

On the other hand, only a small number of immune-associated proteins were found to have higher intensities in the young group relative to the aged group, including M-CSF (CSF-1), CD1d, Galectin1 (LGALS1), CD90 (THY1), and RHOA. Among them, M-CSF (macrophage colony-stimulating factor 1) is a haematopoietic growth factor involved in the proliferation, differentiation, and survival of monocytes and macrophages; in the bone, it is one the most important cytokines for the maturation and function of osteoclasts. CD1d is a key molecule necessary to activate CD1d-dependent natural killer T cells that regulate certain tissue-specific immune activities [65]. Galectin1, a homodimeric galactose-binding lectin, is able to selectively bind with a T cell surface receptor (NP-1) and inhibit T cell proliferation [66, 67]. CD90, also known as the T lymphocyte differentiation antigen, is a conserved cell surface protein with a single V-like immunoglobulin domain; CD90 can activate the T cell receptor [68] and is expressed as a surface marker for bone marrow stem cells [69, 70] and osteoblasts [71].

Collectively, a large number of immune regulatory factors for both innate and adaptive immunity were found in OLCS exosomes. These findings may provide clues for exploring the mutual regulation between the skeleton and immune system and the mechanism of bone ageing.

#### Bone remodelling-associated proteins in OLCS exosomes

Proteins that are involved in osteoblast differentiation (e.g., COL-1/5, LRP, MMP-2, DCN, CX43, β-CATENIN, TGF-β, ALP) and osteoclast differentiation (e.g., M-CSF, ANXA, FBN1, SLC9, SRC, ATP6AP) were found to be upregulated in the young group relative to the aged group (Fig. 7a, c), indicating that exosomes may be one of the possible mechanisms of bone secretion in the regulation of bone remodelling and decrease with age.

#### Antioxidative proteins in OLCS exosomes

Since cell ageing is identified as a decline in various functions of the cell, almost all evidence that supports an increase in harmful effects or a loss of beneficial effects in the aged cell is reasonable. However, there is an exception: some exosomal proteins involved in maintaining cell redox homeostasis and removal of ROS are upregulated in aged OLCS exosomes relative to young ones (Fig. 7b, d), including several key molecules, such as peroxiredoxins (PRDXs), superoxide dismutase 2 (SOD2), thioredoxin 1 (TRX1), glutathione S-transferase P1 (GSTP1), and haptoglobin, which manifests in aged OLCS may exert a positive effect on surrounding or even distant cells to resist oxidative damage and ageing.

## Conclusion

A global view of changes in the gene expression profiles of bone cells and secretory proteins in OLCS exosomes suggests key characteristics of bone ageing. Some changes considered hallmarks of cell ageing have been reviewed previously, e.g., reductions in energy metabolism, cellular responses to hormones, DNA conformation, telomere maintenance, and an increased level of the proinflammatory state. Moreover, newly identified changes are indicative of reductions in bone-specific functions, e.g., mechanical sensation and bone remodelling regulation. Further, our data suggest that exosomes of young OLCS have multiple molecules that could play roles in maintaining cellular homeostasis; however, these functions are weakened during ageing. Nevertheless, the upregulated proteins in aged OLCS exosomes provide clues that these aged OLCS exosomes could have additional functions, such as promoting wound healing and scavenging free radicals, that help the surrounding cells resist senescence. On the other hand, aged cells are considered the principal promoters of systemic and local inflammation that characterize ageing and promote age-related diseases. Herein, we first found that a large number of immune-related DEGs were upregulated in aged bone cells, and then, we determined that many regulatory factors both for innate and adaptive immunity are found in OLCS exosomes, indicating that exosomes may act as shuttles propagating inflammatory states from the aged cells to the immune cells. Taken together, this study provides new insights into a further understanding of bone ageing.

## Additional files

Additional file 1: **Figure S1 and S2.** Description of the sampling method of cortical bone cell in this study. (**Fig. S1.** Cell composition of mouse cortical bone shown by H&E staining. **Fig. S2.** The sampling method of this study). (DOCX 21222 kb)

## Abbreviations

BV/TV: Bone volume to total volume ratio
CBT: Cortical bone thickness
CKD: Chronic kidney disease
CVD: Cardiovascular disease
DAMPs: Damage-associated molecular patterns
DEGs: Differentially expressed genes
DEPs: Differentially expressed proteins
ER: Endoplasmic reticulum
GO: Gene Ontology
KEGG: Kyoto Encyclopedia of Genes and Genomes
LC-MS/MS: Liquid chromatography-mass spectrometry
LCS: Lacunar-canalicular system
LFQ: Label-free quantitation
MSCs: Mesenchymal stem cells
OLCS: Osteocytic lacunar-canalicular system
PAMPs: Pathogen-associated molecular patterns
ROS: Reactive oxygen species
RPKM: Reads per kilobase transcriptome per million reads
SASP: Senescence-associated secretory phenotype
Tb.N: Trabecular number
VECs: Vascular endothelial cells

## References

[1] Kamioka H, Honjo T, Takano-Yamamoto T (2001). A three-dimensional distribution of osteocyte processes revealed by the combination of confocal laser scanning microscopy and differential interference contrast microscopy. Bone..

[2] Knothe Tate ML (2003). “Whither flows the fluid in bone?” An osteocyte’s perspective. J Biomech.

[3] Mcnamara LM, Majeska RJ, Weinbaum S, Friedrich V, Schaffler MB (2010). Attachment of osteocyte cell processes to the bone matrix. Anatomical Record..

[4] Klein-Nulend J, Semeins CM, Ajubi NE, Nijweide PJ, Burger EH (1995). Pulsating fluid flow increases nitric oxide (NO) synthesis by osteocytes but not periosteal fibroblasts--correlation with prostaglandin upregulation. Biochem Biophys Res Commun..

[5] Genetos DC, Kephart CJ, Zhang Y, Yellowley CE, Donahue HJ (2007). Oscillating fluid flow activation of gap junction hemichannels induces ATP release from MLO-Y4 osteocytes. J Cell Physiol..

[6] Cherian PP, Siller-Jackson AJ, Gu S (2005). Mechanical strain opens connexin 43 hemichannels in osteocytes: a novel mechanism for the release of prostaglandin. Mol Biol Cell..

[7] You L, Temiyasathit S, Lee P (2008). Osteocytes as mechanosensors in the inhibition of bone resorption due to mechanical loading. Bone..

[8] Santos A, Bakker AD, Zandieh-Doulabi B, Blieck-Hogervorst JMAD, Klein-Nulend J (2010). Early activation of the beta-catenin pathway in osteocytes is mediated by nitric oxide, phosphatidyl inositol-3 kinase/Akt, and focal adhesion kinase. Biochem Biophys Res Commun.

[9] Rubin J, Rubin C, Jacobs CR (2006). Molecular pathways mediating mechanical signaling in bone. Gene..

[10] Bonewald LF (2007). Osteocytes as dynamic multifunctional cells. Ann N Y Acad Sci..

[11] Marotti G, Ferretti M, Muglia MA, Palumbo C, Palazzini S (1992). A quantitative evaluation of osteoblast-osteocyte relationships on growing endosteal surface of rabbit tibiae. Bone..

[12] Palumbo C, Palazzini S, Marotti G (1990). Morphological study of intercellular junctions during osteocyte differentiation. Bone..

[13] Bellido T, Saini V, Pajevic PD (2013). Effects of PTH on osteocyte function. Bone..

[14] Bonewald LF, Wacker MJ (2013). FGF23 production by osteocytes. Pediatr Nephrol..

[15] Ryan JW, Reinke D, Kogawa M, Turner AG, Atkins GJ, Anderson PH (2013). Novel targets of vitamin D activity in bone: action of the vitamin D receptor in osteoblasts, osteocytes and osteoclasts. Curr Drug Targets..

[16] Dallas SL, Prideaux M, Bonewald LF (2013). The osteocyte: an endocrine cell ... and more. Endocr Rev..

[17] Mizokami A, Kawakubo-Yasukochi T, Hirata M (2017). Osteocalcin and its endocrine functions. Biochemical Pharmacology..

[18] Oury F, Khrimian L, Denny CA, Gardin A, Chamouni A, Goeden N (2013). Maternal and offspring pools of osteocalcin influence brain development and functions. Cell..

[19] Mosialou I, Shikhel S, Liu JM, Maurizi A, Luo N, He Z (2017). MC4R-dependent suppression of appetite by bone-derived lipocalin 2. Nature..

[20] Fitzpatrick EA, Han X, Xiao Z, Quarles LD (2018). Role of fibroblast growth factor-23 in innate immune responses. Frontiers in endocrinology..

[21] Heine GH, Seiler S, Fliser D (2012). Fgf-23: The rise of a novel cardiovascular risk marker in CKD. Nephrology, dialysis, transplantation: official publication of the European Dialysis and Transplant Association. European Renal Association..

[22] Scialla JJ, Wolf M (2014). Roles of phosphate and fibroblast growth factor 23 in cardiovascular disease. Nat Rev Nephrology..

[23] Larsson T, Nisbeth U, Ljunggren O, Juppner H, Jonsson KB (2003). Circulating concentration of FGF-23 increases as renal function declines in patients with chronic kidney disease, but does not change in response to variation in phosphate intake in healthy volunteers. Kidney Int.

[24] Gutierrez O, Isakova T, Rhee E, Shah A, Holmes J, Collerone G (2005). Fibroblast growth factor-23 mitigates hyperphosphatemia but accentuates calcitriol deficiency in chronic kidney disease. J Am Soc Nephrol.

[25] Evenepoel P, Meijers B, Viaene L, Bammens B, Claes K, Kuypers D (2010). Fibroblast growth factor-23 in early chronic kidney disease: additional support in favor of a phosphate-centric paradigm for the pathogenesis of secondary hyperparathyroidism. Clin J Ame Soc Nephrol.

[26] Gonzalez-Garcia ZM, Kullo IJ, Coletta DK, Mandarino LJ, Shaibi GQ (2015). Osteocalcin and type 2 diabetes risk in Latinos: a life course approach. Am J Hum Biol.

[27] Kim GS, Jekal Y, Kim HS, Im JA, Park JY, Chu SH (2014). Reduced serum total osteocalcin is associated with central obesity in Korean children. Obes Res Clin Pract.

[28] Luo Y, Ma X, Hao Y, Xu Y, Xiong Q, Tang J (2015). Association between serum osteocalcin level and visceral obesity in Chinese postmenopausal women. Clin Endocrinol.

[29] Chin KY, Ima-Nirwana S, Mohamed IN, Ahmad F, Ramli ES, Aminuddin A (2014). Serum osteocalcin is significantly related to indices of obesity and lipid profile in Malaysian men. Int J Med Sci.

[30] Wisniewski J, Zougman A, Nagaraj N, Mann M (2009). Universal sample preparation method for proteome analysis. Nature Methods..

[31] Moulder R, Goo YA, Goodlett DR, Sechi S (2016). Label-free quantitation for clinical proteomics. Quantitative proteomics by mass spectrometry.

[32] Yu G, Wang LG, Han Y, He QY (2012). Clusterprofiler: An R package for comparing biological themes among gene clusters. OMICS..

[33] Schneider P, Krucker T, Meyer E, Ulmann-Schuler A, Weber B, Stampanoni M (2009). Simultaneous 3D visualization and quantification of murine bone and bone vasculature using micro-computed tomography and vascular replica. Microsc Res Tech..

[34] Lin C, Jiang X, Dai Z, Guo X, Weng T, Wang J (2009). Sclerostin mediates bone response to mechanical unloading through antagonizing Wnt/beta-catenin signaling. J Bone Miner Res..

[35] Sapir-Koren R, Livshits G (2014). Osteocyte control of bone remodeling: is sclerostin a key molecular coordinator of the balanced bone resorption-formation cycles?. Osteoporos Int..

[36] Haussler MR, Whitfield GK, Kaneko I, Forster R, Saini R, Hsieh J-C (2012). The role of vitamin d in the fgf23, klotho, and phosphate bone-kidney endocrine axis. Rev Endocr Metab Disord.

[37] Nakashima T, Hayashi M, Fukunaga T, Kurata K, Oh-Hora M, Feng JQ (2011). Evidence for osteocyte regulation of bone homeostasis through RANKL expression. Nat Med..

[38] Xiong J, Onal M, Jilka RL, Weinstein RS, Manolagas SC, O'Brien CA (2011). Matrix-embedded cells control osteoclast formation. Nat Med..

[39] Akira S, Uematsu S, Takeuchi O (2006). Pathogen recognition and innate immunity. Cell..

[40] Schindler C, Levy DE, Decker T (2007). JAK-STAT signaling: from interferons to cytokines. Journal of Biological Chemistry..

[41] Lopez-Otin C, Blasco MA, Partridge L, Serrano M, Kroemer G (2013). The hallmarks of aging. Cell..

[42] Bota DA, Davies KJA (2016). Mitochondrial Lon protease in human disease and aging: including an etiologic classification of Lon-related diseases and disorders. Free Radic Biol Med.

[43] Michiorri S, Gelmetti V, Giarda E, Lombardi F, Romano F, Marongiu R (2010). The Parkinson-associated protein PINK1 interacts with Beclin1 and promotes autophagy. Cell Death Differ.

[44] Szabadkai G, Bianchi K, Várnai P, Stefani DD, Wieckowski MR, Cavagna D (2006). Chaperone-mediated coupling of endoplasmic reticulum and mitochondrial Ca2+ channels. J Cell Biol..

[45] de Brito OM, Scorrano L (2008). Mitofusin 2 tethers endoplasmic reticulum to mitochondria. Nature..

[46] Rowland AA, Voeltz GK (2012). Endoplasmic reticulum–mitochondria contacts: function of the junction. Nat Rev Mol Cell Biol.

[47] Eijkelenboom A, Burgering BMT (2013). Foxos: signalling integrators for homeostasis maintenance. Nat Rev Mol Cell Biol..

[48] Almeida M, Han L, Martinmillan M, O'Brien CA, Manolagas SC (2007). Oxidative stress antagonizes Wnt signaling in osteoblast precursors by diverting beta-catenin from T cell factor- to forkhead box O-mediated transcription. J Biol Chem.

[49] Tzivion G, Dobson M, Ramakrishnan G (2011). Foxo transcription factors; regulation by AKT and 14-3-3 proteins. Biochim Biophys Acta..

[50] Coppe JP, Desprez PY, Krtolica A, Campisi J (2010). The senescence-associated secretory phenotype: the dark side of tumor suppression. Annu Rev Pathol.

[51] Freund A, Orjalo AV, Desprez PY, Campisi J (2010). Inflammatory networks during cellular senescence: causes and consequences. Trends Mol Med.

[52] Chen C, Liang MK, Zhang H, Peng YQ, Wu XP, Wu XY (2014). Relationships between age-related biochemical markers of bone turnover and OPG, TGF-beta1 and TGF-beta2 in native Chinese women. Endocr Res..

[53] Hatori K, Sasano Y, Takahashi I, Kamakura S, Kagayama M, Sasaki K (2004). Osteoblasts and osteocytes express MMP2 and -8 and TIMP1, -2, and -3 along with extracellular matrix molecules during appositional bone formation. Anat Rec A Discov Mol Cell Evol Biol..

[54] Khokha R, Murthy A, Weiss A (2013). Metalloproteinases and their natural inhibitors in inflammation and immunity. Nat Rev Immunol.

[55] Salminen A, Kaarniranta K (2011). Control of p53 and NF-kappa B signaling by WIP1 and MIF: role in cellular senescence and organismal aging. Cell Signal..

[56] Hughes DE, Salter DM, Simpson R (1994). Cd44 expression in human bone: a novel marker of osteocytic differentiation. J Bone Miner Res..

[57] Günthert U, Schwärzler C, Wittig B, Laman J, Ruiz P, Stauder R (1998). Functional involvement of CD44, a family of cell adhesion molecules, in immune responses, tumour progression and haematopoiesis. Oxygen Trans Tiss XXXIII.

[58] Haynes BF, Telen MJ, Hale LP, Denning SM (1989). CD44--a molecule involved in leukocyte adherence and T-cell activation. Immunology Today..

[59] Gardai SJ, Mcphillips KA, Frasch SC, Janssen WJ, Starefeldt A, Murphyullrich JE (2005). Cell-surface calreticulin initiates clearance of viable or apoptotic cells through trans-activation of LRP on the phagocyte. Cell..

[60] Brown EJ, Frazier WA (2001). Integrin-associated protein (CD47) and its ligands. Trends Cell Biol.

[61] Maile LA, Demambro VE, Wai C, Aday AW, Capps BE, Beamer WG (2011). An essential role for the association of CD47 to SHPS-1 in skeletal remodeling. J Bone Min Res.

[62] Bloom AC, Collins FL, Van’t Hof RJ, Ryan ES, Jones E, Hughes TR (2016). Deletion of the membrane complement inhibitor CD59a drives age and gender-dependent alterations to bone phenotype in mice. Bone..

[63] Travis MA, Sheppard D (2014). Tgf-β activation and function in immunity. Ann Rev Immunol.

[64] Tang SY, Alliston T (2013). Regulation of postnatal bone homeostasis by tgf[beta]. Bonekey Rep..

[65] Godfrey DI, Macdonald HR, Kronenberg M, Smyth MJ, Van KL (2004). Nkt cells: what’s in a name?. Nat Rev Immunol..

[66] Catalano A, Caprari P, Moretti S, Faronato M, Tamagnone L, Procopio A (2006). Semaphorin-3a is expressed by tumor cells and alters T-cell signal transduction and function. Blood..

[67] Kikutani H, Kumanogoh A (2003). Semaphorins in interactions between T cells and antigen-presenting cells. Nat Rev Immunol..

[68] Haeryfar SM, Hoskin DW (2004). Thy-1: More than a mouse pan-T cell marker. J Immunol.

[69] Boxall SA, Jones E (2012). Markers for characterization of bone marrow multipotential stromal cells. Stem Cells Int..

[70] Peister A, Mellad JA, Larson BL, Hall BM, Gibson LF, Prockop DJ (2004). Adult stem cells from bone marrow (MSCs) isolated from different strains of inbred mice vary in surface epitopes, rates of proliferation, and differentiation potential. Blood..

[71] Chen XD, Qian HY, Neff L, Satomura K, Horowitz MC (2010). Thy-1 antigen expression by cells in the osteoblast lineage. J Bone Min Res.

