# Supplementary material for: Ageing characteristics of bone indicated by transcriptomic and exosomal proteomic analysis of cortical bone cells
Source: J Orthop Surg Res. 2019 May 10;14:129. doi: 10.1186/s13018-019-1163-4 (PMC6509863; doi:10.1186/s13018-019-1163-4)
Supplement: Supplementary file 1 — Figure S1 and S2. Description of the sampling method of cortical bone cell in this study. (Fig. S1. Cell composition of mouse cortical bone shown by H&E staining. Fig. S2. The sampling method of this study). (DOCX 21222 kb) [file 13018_2019_1163_MOESM1_ESM.docx]

**Characteristics of osteocyte ageing indicated by transcriptomic and exosomal proteomic analysis of cortical bone**

Additional file


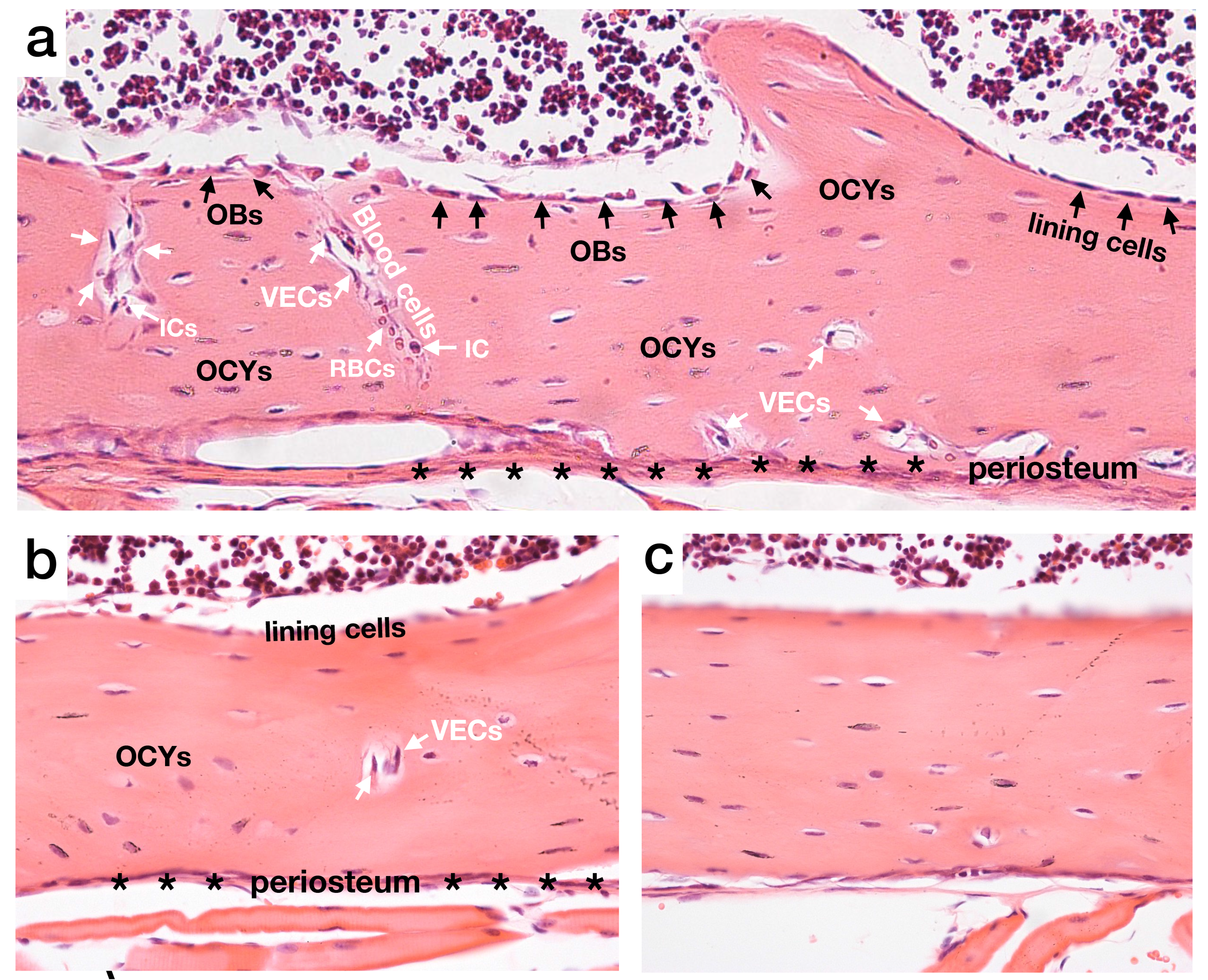


**Fig. S1. Cell composition of mouse cortical bone shown by H&E staining.**

(a) Cortical bone in the distal metaphyseal mouse femur. The black arrows indicate osteoblasts (OBs) and bone lining cells, white arrows indicate blood vessels and blood cells, including vascular endothelial cells (VECs), inflammatory cells (ICs) and red blood cells (RBCs). Black stars indicate the bone periosteum. Osteocytes (OCYs) are ubiquitous in the bone matrix.

(b) Cortical bone in the mid-shaftmouse femur (the sampling site of this study), where osteocytes are ubiquitous and blood vessels are less distributed.


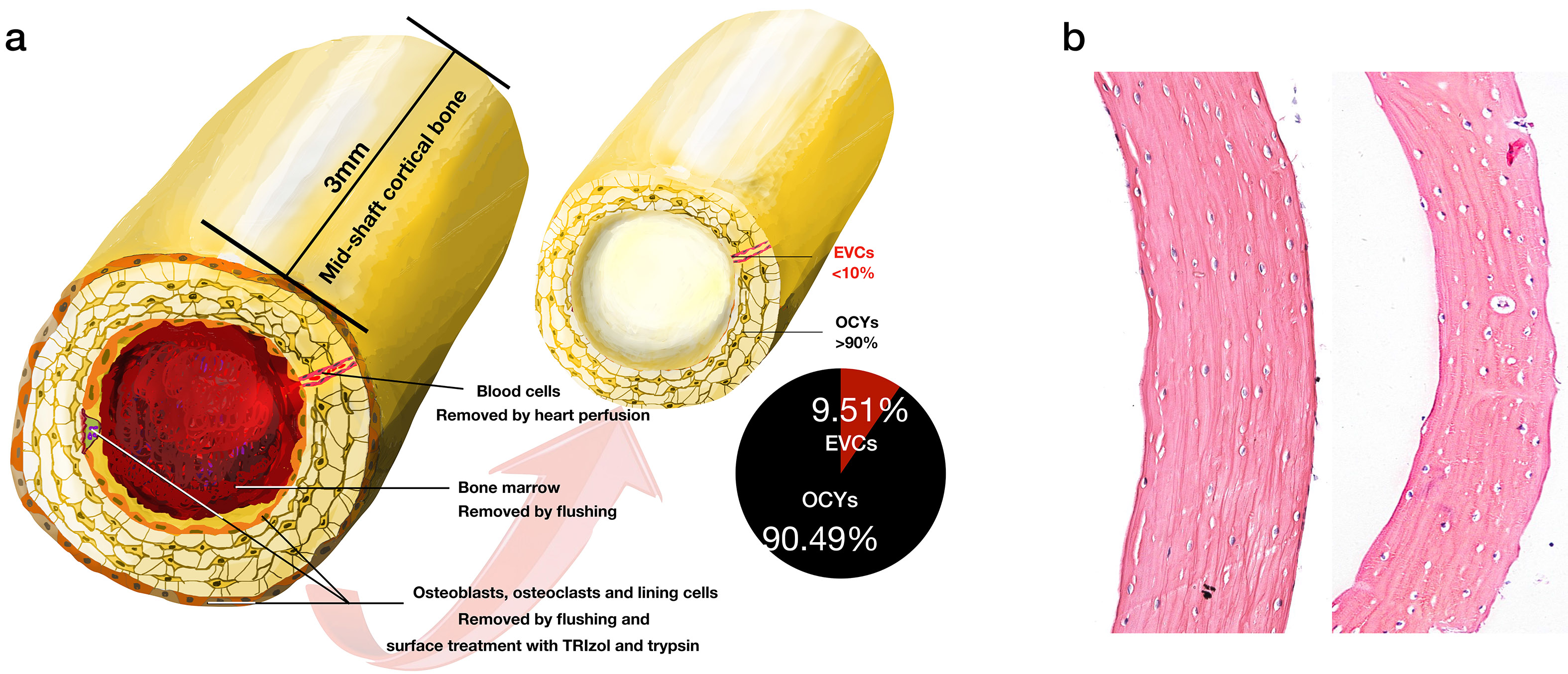


**Fig. S2. The sampling method of this study.**

(a) The following methods were used to ensure that osteocytes were dominant in the sampling site. By heart perfusion, most blood cells can be removed from the bone. Then, the 3-mm section of mid-shaft cortical bone was amputated. By flushing and surface treatment with PBS, TRIzol and typsin, most of the adherent cells can be removed, including osteoclasts (OCs), osteoblasts (OBs), lining cells and periosteum cells. In the preliminary experiment, horizontal serial sections of mouse femur cortical bone were made, 22 sections were randomly selected for cell counting. The ratio of VECs and osteocytes in the sections was calculated. It showed that the proportion of osteocytes was 90.49%, indicating that most of the components obtained by this sampling method are contributed by osteocytes.

(b) H&E stained horizontal sections of mouse femur cortical bone after surface treatment. The mid-shaft cortical bone pretreated by surface flushing with PBS (the figure on the left), and by further short digestion with trypsin (the figure on the right).

Illustration：After surface treatment, bone marrow and adherent cells can be mostly removed. Osteocytes comprise the overwhelming majority of cells in the sampling site.
